# Editorial: Biomarkers of Radio-Sensitivity and Radio-Resistance

**DOI:** 10.3389/fonc.2018.00076

**Published:** 2018-03-19

**Authors:** Roberta Di Pietro

**Affiliations:** ^1^Department of Medicine and Aging Sciences, G. d’Annunzio University of Chieti-Pescara, Chieti-Pescara, Italy

**Keywords:** radiation effects, radiosensitivity, radioresistance, radiotherapy, biomarkers

**Editorial on the Research Topic**

**Biomarkers of Radio-Sensitivity and Radio-Resistance**

In the era of precision medicine, radiation oncologists have improved the outcomes of personalized treatments thank to technological advances obtained in the past few decades and to novel biological concepts leading to a biomarker-guided prescription and follow-up. The concept of precision medicine—prevention and treatment strategies tailored to individual patient—is not new (1), but the broad application of this concept has been recently favored by the availability of large-scale biologic databases (like the human genome sequence), computational tools for analyzing large sets of data, and improved methods for characterizing patients (like proteomics, metabolomics, genomics, multiple cellular assays, and even mobile health technology) (2). However, in spite of the great efforts made by the scientific community, healthcare workers and patients, there are still a number of obstacles to be overcome along the path toward precision oncology: unexplained drug resistance, genomic and histologic heterogeneity of tumors, insufficient clinical trials for monitoring responses and tumor recurrence, and limited knowledge about the use of drug combinations (2). Furthermore, the high specialization required and the high cost of sophisticated technologies limit both the number of excellent centers for individualized treatments and the access to therapy of large populations fringes, making the fate different between rich and poor people.

In the attempt to spread the current knowledge about biomarkers of radio-sensitivity and radio-resistance, this topic issue has taken into account both the mechanisms of cell response to radiation and the mechanisms of repair of damage following ionizing radiation therapy. In particular, in the mini-review “Regulation of Cancer Cell Responsiveness to Ionizing Radiation Treatment by Cyclic AMP Response Element Binding Nuclear Transcription Factor”. Francesca D’Auria et al. focus their attention on the CREB pathway in solid tumors treated with radiotherapy (RT), including hematological, gastrointestinal, lung, and prostate cancer. The authors clearly illustrate the crosstalk between CREB/ATF-family proteins and other signaling molecules involved in stress response and highlight the pleiotropic action of CREB in mediating cell death or survival in irradiated tumor cells, concluding that further preclinical work is still necessary to identify and better characterize the molecular networks involved in this important hub before going into clinical trials.

In the mini-review “DNA Repair and Cytokines: TGF-β, IL-6, and Thrombopoietin as Different Biomarkers of Radioresistance,” Centurione and Aiello, after describing in an exhaustive way the different DNA repair pathways activated in response to irradiation, report the activity of cytokines on DNA repair suggesting that RT effects can be improved by means of cytokines-targeted therapies. In particular, they show the mechanisms through which TGF-β and IL-6 confer radio-resistance and the ways to counteract this effect with specific inhibitors before RT administration.

The next two articles of this topic issue are more focused on clinical aspects. Chin et al. in “Hyperspectral Imaging as an Early Biomarker for Radiation Exposure and Microcirculatory Damage” propose the post-exposure measurement of cutaneous deoxygenated hemoglobin levels as a useful biomarker for radiation dose reconstruction and as a predictor for chronic micro-vascular injury. The authors established a reliable model of radiation-induced skin injury using a hairless mouse and a single fixed-dose of surface beta-irradiation. They also demonstrate how it is possible to identify characteristic changes in cutaneous perfusion prior to the visible appearance of skin injury by means of hyperspectral imaging (HSI) technology, in order to avoid undesirable treatment breaks that could negatively impact recurrence rate and overall survival.

In the last review article, “Radiation Metabolomics: Current Status and Future Directions” Menon et al. report in a comprehensive way the state of the art and perspectives of radiation metabolomics highlighting the advantages of this approach compared to other less recent “omics” like genomics, transcriptomics, and proteomics. The authors clearly outline this emerging field of research and analyze its integration in systems biology with the aim to identify specific pathways that can be targeted to alleviate or mitigate side effects of radiation exposure.

Finally, I would like to thank all the authors for their excellent contributions as well as the Editorial Board and all the voluntary referees for their generous and competent help in the editing process. I do hope that this topic issue will be of interest to a broad audience and useful to the scientific community to make progress in identifying new more accurate and reliable signatures of responsiveness or unresponsiveness to radiation therapy, with the ultimate goal to be able to administrate the right radiation dose, at the right moment to the right patient.

## Author Contributions

The author confirms being the sole contributor of this work and approved it for publication.

## Conflict of Interest Statement

The author declares that the research was conducted in the absence of any commercial or financial relationships that could be construed as a potential conflict of interest.

## References

[1] National Research Council. Toward Precision Medicine: Building a Knowledge Network for Biomedical Research and a New Taxonomy of Disease. Washington, DC: National Academies Press (2011). 22536618

[2] Collins FS Varmus H. A new initiative on precision medicine. N Engl J Med (2015) 372:793–5. 10.1056/NEJMp1500523 25635347 PMC5101938

